# The Beneficial Effects of Stingless Bee Honey from *Heterotrigona itama* against Metabolic Changes in Rats Fed with High-Carbohydrate and High-Fat Diet

**DOI:** 10.3390/ijerph16244987

**Published:** 2019-12-07

**Authors:** Nur Zuliani Ramli, Kok-Yong Chin, Khairul Anwar Zarkasi, Fairus Ahmad

**Affiliations:** 1 Department of Anatomy, Faculty of Medicine, UKM Medical Centre, Universiti Kebangsaan Malaysia, Cheras 56000, Kuala Lumpur, Malaysia; 2 Department of Biomedical Sciences and Therapeutics, Faculty of Medicine and Health Sciences, Universiti Malaysia Sabah, Jalan UMS, Kota Kinabalu 88400, Sabah, Malaysia; nurzuliani@ums.edu.my; 3 Department of Pharmacology, Faculty of Medicine, UKM Medical Centre, Universiti Kebangsaan Malaysia, Cheras 56000, Kuala Lumpur, Malaysia; chinkokyong@ppukm.ukm.edu.my; 4 Department of Biochemistry, Faculty of Medicine, UKM Medical Centre, Universiti Kebangsaan Malaysia, Cheras 56000, Kuala Lumpur, Malaysia; khairul.anwar@ums.edu.my

**Keywords:** metabolic syndrome, central obesity, hypertriglyceridemia, high blood pressure, Kelulut honey, adipocyte hypertrophy, liquid chromatography-mass spectrometry

## Abstract

Metabolic syndrome (MetS) is a group of conditions including central obesity, hyperglycemia, dyslipidemia, and hypertension that increases the risk for cardiometabolic diseases. Kelulut honey (KH) produced by stingless honey bees has stronger antioxidant properties compared to other honey types and may be a functional food against MetS. This study aimed to determine the efficacy of KH in preventing metabolic changes in rats with MetS induced by high-carbohydrate and high-fat (HCHF) diet. Male Wistar rats were randomly assigned to the control (C), HCHF diet-induced MetS (S), and MetS supplemented with KH (K) groups. The K group was given KH (1 g/kg/day) for eight weeks. Compared to the control, the S group had significant higher omental fat mass, serum triglyceride, systolic blood pressure, diastolic blood pressures, adipocyte area, and adipocyte perimeter (*p* < 0.05). KH supplementation significantly prevented these MetS-induced changes at week 16 (*p* < 0.05). Several compounds, including 4-hydroxyphenyl acetic acid, coumaric and caffeic acids, had been detected via liquid chromatography-mass spectrometry analysis that might contribute to the reversal of these changes. The beneficial effects of KH against MetS-induced rats provide the basis for future KH research to investigate its potential use in humans and its molecular mechanisms in alleviating the disease.

## 1. Introduction

Metabolic syndrome (MetS) is a group of risk factors that increase the tendency for cardiovascular diseases (CVD) and diabetes mellitus [1]. Globally, the prevalence of MetS varies by age, gender, and ethnicity, whereby individuals over the age of 50 years old, women, and those from Indian descent are at a greater risk of developing the syndrome [2]. In the United States from 2003 to 2012, approximately 33% of the population over the age of 20 years old have been reported to have MetS [3]. On the other hand, the prevalence of MetS in the Asia Pacific region in 2017 was between 11.9% and 37.1%. Among the Southeast Asian countries, Malaysia had the highest prevalence of MetS (34.3%) compared to the Philippines (11.9%), Indonesia (28.4%), and Singapore (20.2%) [4]. Other factors that could increase the incidence of MetS are city-dwellers, rapid urbanization, and sedentary lifestyle especially among developing countries [2,5]. In addition, a diet containing high levels of saturated fat with low fiber, as well as obesity (both general and central obesity) also contribute to the occurrence of MetS [6].

Current MetS management strategies involve adopting an active lifestyle and intake of a balanced diet, followed by pharmaceutical therapy if the lifestyle modifications were proven to be inadequate [7]. However, patients face several challenges while undergoing treatment. These include difficulty to comply with the medication schedule, the occurrence of medication-related side effects, drug-to-drug interactions, as well as the need for continuous clinic visits which indirectly increase individual and national healthcare cost [8]. Hence, preventive measures are the best strategies for dealing with MetS.

In relation to that, the potential use of natural products for the management and prevention of MetS is actively being studied [9], which include natural honey [10,11]. Studies performed both in vitro and in vivo using several types of honey such as Tualang, Gelam, mad, and honeydew have been reported to exhibit protection against various MetS components attributed by their antioxidative and anti-inflammatory [12]; hypoglycemic and hypolipidemic [13]; pancreatic protective [14]; hepatoprotective [15]; anti-hypertensive [16]; and anti-obesity properties [17,18].

Kelulut honey (KH) is produced by stingless bees found in Malaysia. In contrast to other local honey species, KH has been reported to possess stronger antioxidant activity and higher phenolic content [19,20]. It also has the potential to be used in the treatment of various medical conditions due to its anti-bacterial, anti-fungal and anti-cancer properties [21,22,23]. However, the effects of KH on MetS are still unknown. Therefore, this study was performed to determine the effects of KH on rats with MetS induced by high-carbohydrate and high-fat (HCHF) diet.

## 2. Materials and Methods

### 2.1. Kelulut Honey Preparation

Raw KH was harvested from stingless honey bee species of *Heterotrigona itama* from a local honey bee farm (Gombak, Selangor, Malaysia). The honey was stored in a glass jar at 4 °C until further use. Upon administration to rats, KH was diluted with distilled water at a 1:1 ratio.

### 2.2. Liquid Chromatography-Mass Spectrometry (LC-MS) Analysis of Kelulut Honey

Identification of the bioactive compounds in KH was performed using SCIEX 3200QTRAP^®^ hybrid trap mass spectrometer (AB SCIEX, Massachusetts, USA) coupled with PerkinElmer^®^ Flexar^®^ FX-15 UHPLC (PerkinElmer, Massachusetts, USA). The column used was a reversed-phase Phenomenex Synergi RP C18, 100 Å (pore size), 100 mm (length) × 2.0 mm (diameter) × 3 µM (particle size) (Phenomenex, California, USA). KH (1 mL) was dissolved in ultrapure water (1 mL) (Fischer Scientific, New Hampshire, USA), filtered through a 0.45 µm nylon syringe filter (Minisart^®^ NY, Sartorius Stedim Biotech, Göttingen, Germany) to remove impurities before being injected into the LC-MS/MS system. The conditions were set as follows: Solvent A– water with 0.1% formic acid (Fischer Scientific, New Hampshire, USA) and 5 mM ammonium formate (Fischer Scientific, New Hampshire, USA), solvent B – acetonitrile (Fischer Scientific, New Hampshire, USA) with 0.1% formic acid and 5 mM ammonium formate, elution gradient program was set as followed: 0.1 min, 95% A; 10 min, 0% A; 12.1 min, 95% A; then equilibrated for 3 min (total run time – 15 min), flow rate at 0.4 mL/min, injection volume of 20 µL, ESI parameters in negative ion mode, and mass range from 100–1500 m/z. The instrumental parameters were set as follows: Nebulizer gas GS1 pressure at 40 pounds per square inch gauge (psig) and GS2 pressure at 30 psig, desolvation temperature (TEM) at 500 °C, collision activated dissociation gas (CAD) at -3 arbitrary unit (AU) and ionspray voltage (IS) at −4500 V. The data acquisition and processing were carried out using Analyst version 1.5.2 software (AB SCIEX, Massachusetts, USA). Compound identification was performed by cross-referencing both the internal and online databases including Human Metabolome Database [24], Metlin [25], Biological Magnetic Resonance Data Bank [26], and mzCloud [27].

### 2.3. Animals

This experiment has been approved by the Universiti Kebangsaan Malaysia (UKM) Animal Ethical Committee (approval no. ANAT/PP/2017/FAIRUS AHMAD/27-SEPT. /871-OCT.-2017-SEPT.-2018). A total of 18 male Wistar rats weighed 250–300 g and aged three months old were purchased from the Laboratory Animal Resource Unit, UKM (Kuala Lumpur, Malaysia). The rats were acclimatized for two weeks and housed individually in the Animal Laboratory of the Anatomy Department, UKM (Cheras, Malaysia). The ambient temperature was maintained at 25 ± 3 °C with good air ventilation and 12 h of the light/dark cycle.

### 2.4. Study Design

The rats were randomly assigned into three groups (*n* = 6 for each group) comprising of healthy control (C), MetS-induced by HCHF diet (S), and MetS treated with KH (K) groups. Rats in the C group were fed with standard rat chow and tap water while rats in both S and K groups received HCHF diet with 25% fructose drinking water for 16 weeks. Additionally, the K group was administered with KH at the dose of 1 g/kg/day via oral gavage during the last eight weeks of the experiment while vehicle solution (distilled water) was replaced for both C and S groups [14].

### 2.5. High-Carbohydrate and High-Fat Diet Preparation

The high-carbohydrate and high-fat (HCHF) diet was prepared by mixing 175 g fructose [D-(-)-Fructose Emprove^®^ Essential, Merck, Darmstadt, USA), 395 g sweetened condensed milk (Fraser & Neave Holdings Bhd., Kuala Lumpur, Malaysia), 200 g ghee (Enrico’s Pure Ghee, Raviraj Sdn. Bhd., Penang, Malaysia), 25 g Hubble, Mendel, and Wakeman salt mixture (MP Biomedicals, California, USA), 155 g powdered rat chow (Gold Coin Feedmills (M) Sdn. Bhd., Selangor, Malaysia) and 50 g water, in addition to 25% fructose (Merck) drinking water according to the method by Wong and colleague [28]. Food and water were given ad libitum.

### 2.6. Measurement of Body Weight 

Body weight of the rats was measured by using a digital weighing scale (Nimbus^®^ Precision Balances, Adam Equipment, Buckinghamshire, UK) at baseline, 8th and 16th week of the study period.

### 2.7. Physiological Parameters

The rats were fasted for 12 h while fructose drinking water was replaced with tap water overnight. Then, oral glucose tolerance test (OGTT) was done after determining the overnight fasting blood glucose level [29]. Following administration of 2 g/kg of 40% glucose solution [D(+)-glucose, ChemPur^®^ Systerm^®^, Classic Chemicals Sdn. Bhd., Malaysia] via oral gavage, blood glucose was measured from rat’s tail vein at 30, 60, 90, and 120 min using Accu-Chek^®^ Performa 2 glucometer (Roche Diagnostics, Switzerland). Systolic (SBP) and diastolic blood pressure (DBP) were measured by using the CODA^®^ tail-cuff blood pressure system (Kent Scientific Corporation, Torrington, CT, USA). Before taking measurements, rats were firstly acclimatized to the CODA^®^ tail-cuff blood pressure system for 10 min and the rat’s tail temperature was ensured to be between 32 °C and 35 °C.

### 2.8. Serum Fasting Lipid Profile Levels Measurement

Blood was obtained from the tail vein of the anesthetized rats at baseline, week 8, and 16. Before blood collection, the rats were fasted overnight and the fructose drinking water was replaced with tap water. For serum extraction, the blood was left to clot at room temperature for 20 min and subsequently centrifuged at 4000 rpm for 30 min. Determination of fasting serum lipid profile levels that included total cholesterol (TC), triglyceride (TG), high-density lipoprotein (HDL), and low-density lipoprotein (LDL) was done using automated clinical chemistry system model Dimension^®^ Xpand^®^ Plus (Siemens AG, Munich, Germany).

### 2.9. Serum Insulin Level Measurement

Serum insulin levels were measured at baseline and week 16 by using enzyme-linked immunosorbent (ELISA) technique with RatINS (Insulin) ELISA kit (Elabscience, Wuhan, China). The optical density was determined via Multiskan^™^ GO Microplate Spectrophotometer (Thermo Scientific, Massachusetts, USA) at the wavelength of 450 nm.

### 2.10. Body Composition Measurements

For body composition, dual-energy X-ray absorptiometry (DXA) scans were performed using Hologic Discovery Densitometer (Hologic Inc., Massachusetts, USA) with Small Animal Analysis Software, Hologic QDR-1000 System, at baseline, week 8, and 16. Rats were anesthetized with KTX during the DXA scan procedure. The whole-body scan provided measurements of fat percentage and lean mass.

### 2.11. Gross and Histomorphometry of the Adipose Tissue

At the end of week 16, the rats were sacrificed by cervical dislocation following anesthesia. A mid-ventral abdominal incision was performed, and the visceral adipose tissue deposition was observed. The omental adipose tissue (attached to the stomach) was then dissected, weighed, and preserved in a 10% buffered formalin. Subsequently, the tissue was processed, embedded in paraffin wax, and sectioned (6 µm thick) before hematoxylin and eosin staining. The stained tissue section was mounted on a glass slide and viewed under a light microscope with 400× total magnification. Photomicrographs of adipocytes were taken using camera (Leica, Wetzlar, Germany) while CaseViewer version 2.2 software (3DHISTECH Ltd., Budapest, Hungary) was used to analyze the histomorphometry of the adipose tissue. The measuring area of 350 µm × 250 µm was predetermined in each sample using “Free Size Rectangle Annotations” tool before the histomorphometry analysis was performed. The number of adipocytes within the measuring area was counted excluding the cells that were at the border. To measure the adipocyte size, the “Closed Polygon Annotations” tool were used to trace the individual adipocyte border. This automatically generated the area and perimeter of the adipocytes.

### 2.12. Statistical Analysis

All parameters were analyzed using SPSS^®^ software version 23 (IBM, Armonk, USA). Normality of the data was assessed using Shapiro-Wilk test. Normally distributed data were analyzed by using mixed-design analysis of variance (ANOVA) with small effect analysis as post hoc test and presented as mean ± standard error of the mean (SEM). Skewed data were compared using Kruskal Wallis test and Mann Whitney U-test with Bon Ferroni adjustment and presented as the median and interquartile range (IQR).

## 3. Results

### 3.1. Analysis of Kelulut Honey by LC-MS

The compounds detected in KH are 4-hydroxyphenyl acetic acid; caffeic acid derivative; caffeoyl glucose derivative; caffeoyl hexoside derivative; coumaric acid; gluconic acid; kynurenic acid derivative; pinobanksin; quinic acid; niazimicin; bisosthenon b; (6β,7α,12β,13β)-7-hydroxy-11,16-dioxo-8,14-apianadien-22,6-olide; aegle marmelos alkaloid c; 7-chloro-6-demethylcepharadione B; n-acetylglycine; and lanosterol (Table 1).

### 3.2. Changes in the Body Weight

**Figure 1:**
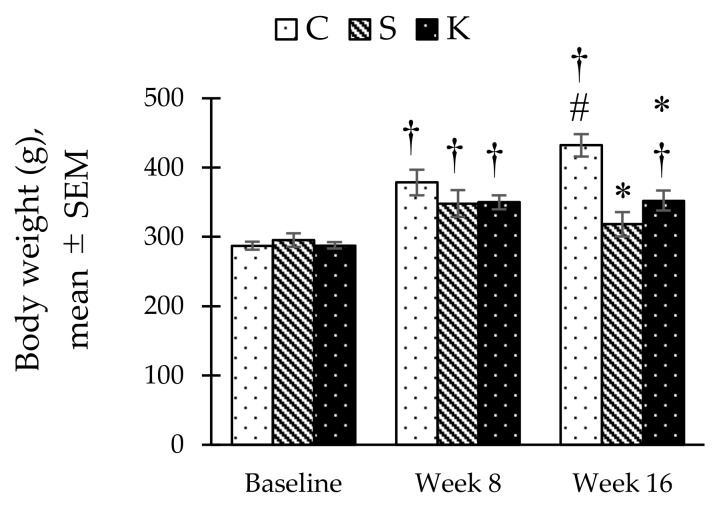
Bar chart showing changes in the body weight in C, S, and K rats. * Significant difference compared to C within the same week, *p* < 0.05. ^#^ Significant difference within the same group at week 16 compared to week 8, *p* < 0.05. ^†^ Significant difference within the same group compared to baseline, *p* < 0.05. The error bars represent the standard error of mean with *n* = 6 in each group. Abbreviation: C (control), S (HCHF diet-induced MetS), K (MetS + Kelulut honey).

Body weight increased over time in all study groups, however this body weight gain was significantly lower in S rats compared to the control at week 16. Although KH supplementation appeared to attenuate weight loss in the K group compared to the S group, this effect was not statistically significant (352.42 ± 14.57 g vs. 318.10 ± 17.58 g, *p* = 0.153) (Figure 1).

### 3.3. Changes in the Serum Insulin and Oral Glucose Tolerance Test

**Figure 2:**
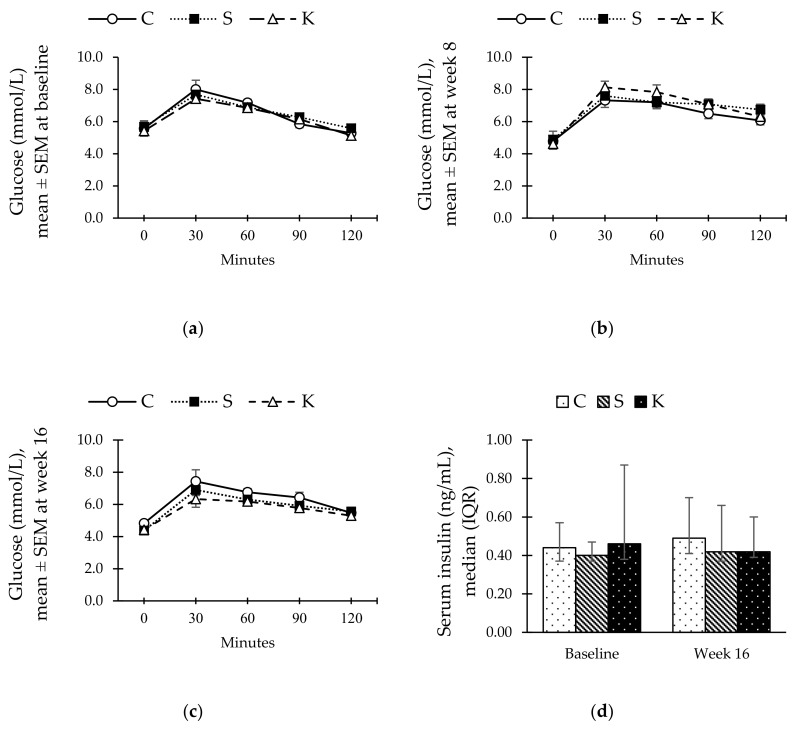
Charts showing changes in glucose curve of oral glucose tolerance test (OGTT) at (**a**) baseline, (**b**) week 8, and (**c**) week 16, as well as (**d**) serum insulin in C, S, and K rats. The error bars represent the standard error of mean with *n* = 6 in each group. Abbreviation: C (control), S (HCHF diet-induced MetS), K (MetS + Kelulut honey).

The rats in the S and K groups demonstrated no significant changes in their OGTT readings as compared to the C rats (Figure 2a–c). Similarly, HCHF diet did not cause any changes in serum insulin level in both the S and K groups (Figure 2d).

### 3.4. Changes in the Systolic and Diastolic Blood Pressure

**Figure 3:**
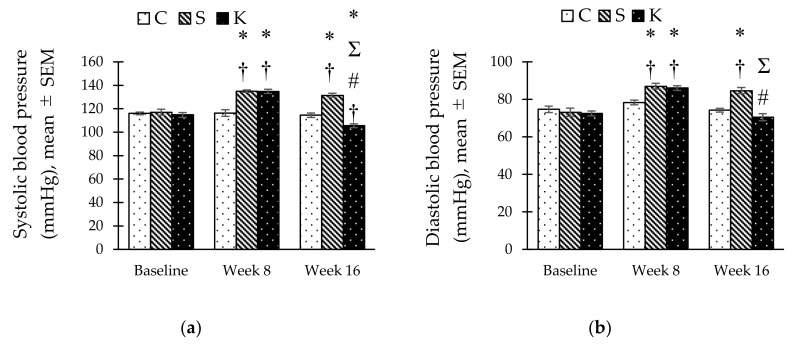
Bar chart showing changes in (**a**) systolic blood pressure and (**b**) diastolic blood pressure in C, S, and K rats. * Significant difference compared to C within the same week, *p* < 0.05. ^#^ Significant difference within the same group at week 16 compared to week 8, *p* < 0.05. ^Ʃ^ Significant difference compared to S within the same week, *p* < 0.05. ^†^ Significant difference within the same group compared to baseline, *p* < 0.05. The error bars represent the standard error of mean with *n* = 6 in each group. Abbreviation: C (control), S (HCHF diet-induced MetS), K (MetS + Kelulut honey).

HCHF diet caused an increase in SBP in the S and K groups as early as week 8 compared to the C group (Figure 3a). SBP in the S group remained significantly higher than the C group at the end of week 16 (131.50 ± 1.65 vs. 114.50 ± 1.96 mmHg, *p* < 0.001) while the reading was lower in the K group (105.50 ± 1.57 mmHg, *p* = 0.001). Similarly, the DBP of the S and K groups was increased significantly after MetS induction at week 8 and maintained significantly higher at week 16 for the S group compared to C group (84.67 ± 1.63 vs. 74.17 ± 1.01 mmHg, *p* < 0.001). Conversely, KH supplementation significantly reduced the DBP reading in the K group compared to S group (70.50 ± 1.88 vs. 84.67 ± 1.63 mmHg, *p* < 0.001) at the end of week 16 (Figure 3b).

### 3.5. Changes in the Fasting Serum Lipid Profile

**Figure 4:**
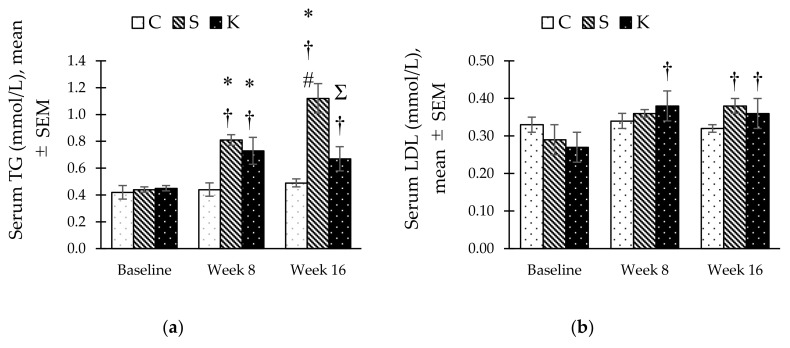

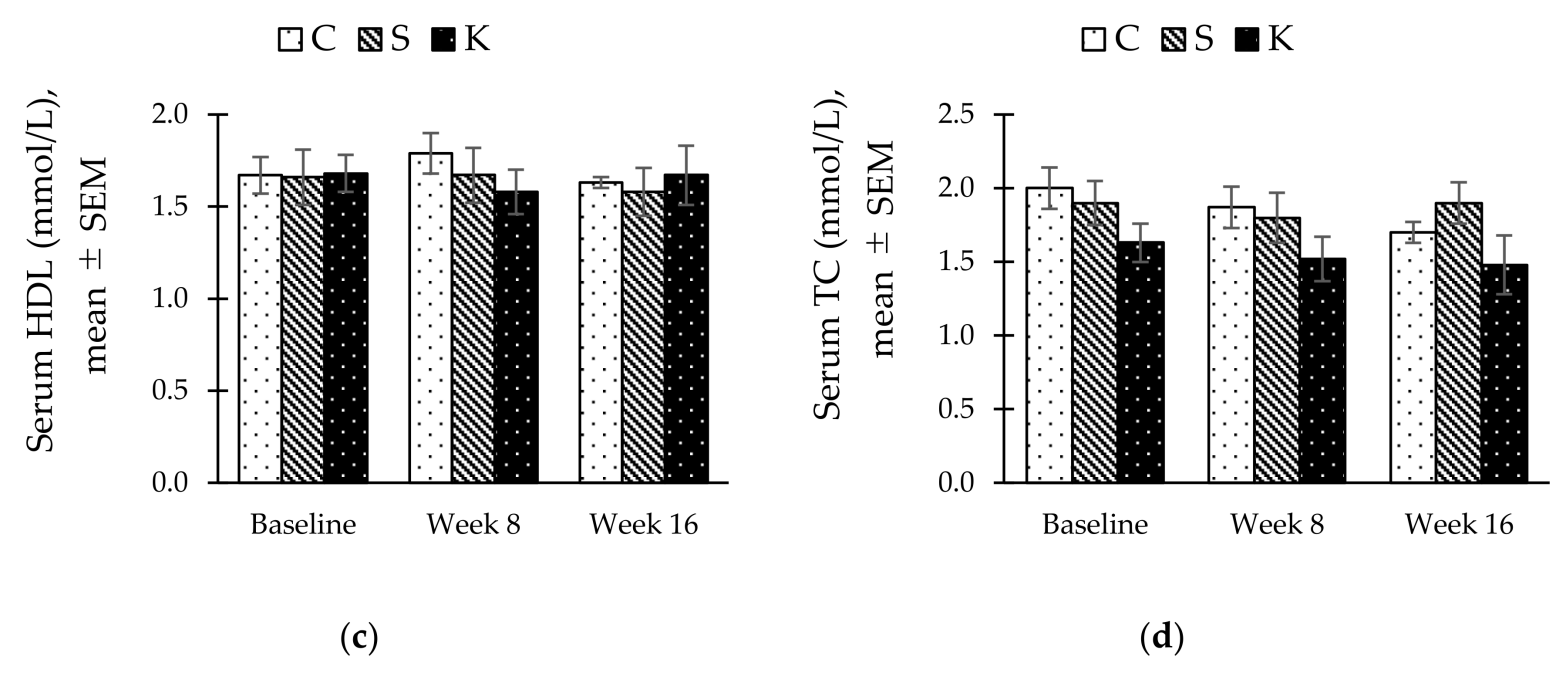
Bar chart showing changes in (**a**) serum triglyceride (TG), (**b**) serum low-density lipoprotein (LDL), (**c**) serum high-density lipoprotein (HDL) and, (**d**) serum total cholesterol (TC) in C, S, and K rats. * Significant difference compared to C within the same week, *p* < 0.05. ^#^ Significant difference within the same group at week 16 compared to week 8, *p* < 0.05. ^Ʃ^ Significant difference compared to S within the same week, *p* < 0.05. ^†^ Significant difference within the same group compared to baseline, *p* < 0.05. The error bars represent the standard error of mean with *n* = 6 in each group. Abbreviation: C (control), S (HCHF diet-induced MetS), K (MetS + Kelulut honey).

The S group showed significant two-fold increase in serum TG levels at week 8 compared to its baseline level and continued to rise significantly compared to the C rats at week 16 (1.12 ± 0.11 vs. 0.49 ± 0.03 mmol/L, *p* < 0.001) (Figure 4a). Contrarywise, KH supplementation caused a reduction in TG levels in the K group compared to the S group (0.67 ± 0.09 mmol/L, *p* = 0.002) at week 16. There was a significant increase in serum low-density lipoprotein (LDL) in MetS-induced rats (both S and K) at week 16 compared to the same groups at baseline. Unlike serum TG, KH supplementation did not prevent the elevation of serum LDL (Figure 4b). Additionally, neither MetS induction nor KH supplementation altered significantly the HDL and TC levels (Figure 4c–d).

### 3.6. Changes in the Body Composition

**Figure 5:**
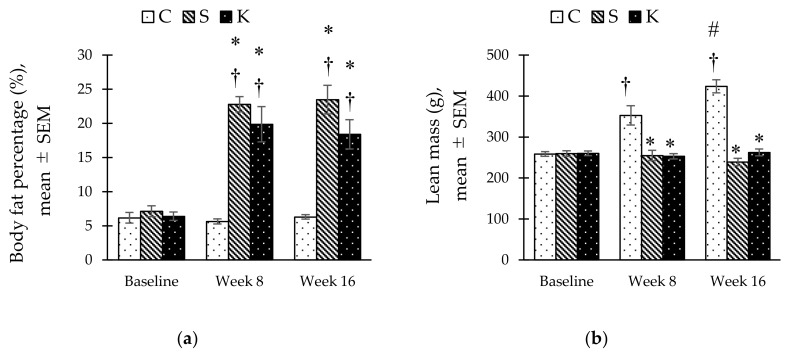
Bar chart showing changes in (**a**) body fat percentage and, (**b**) lean mass in C, S, and K rats. * Significant difference compared to C within the same week, *p* < 0.05. ^#^ Significant difference within the same group at week 16 compared to week 8, *p* < 0.05. ^†^ Significant difference within the same group compared to baseline, *p* < 0.05. The error bars represent the standard error of mean with *n* = 6 in each group. Abbreviation: C (control), S (HCHF diet-induced MetS), K (MetS + Kelulut honey).

Body fat percentage in the S group increased significantly at week 8 and 16 compared to baseline (Figure 5a). With KH supplementation, body fat percentage of the K group at week 16 was reduced as much as 21.6% compared to the S group (18.40 ± 2.15% vs. 23.48 ± 2.10%, *p* = 0.057). Meanwhile, the C group had a significantly higher lean mass than the S and K groups at week 8 and week 16. KH supplementation did not alter the lean mass in the K group compared to the S group (262.24 ± 8.41 g vs. 253.10 ± 6.57, *p* = 0.184) (Figure 5b).

### 3.7. Changes in the Gross Adipose Tissue

**Figure 6:**
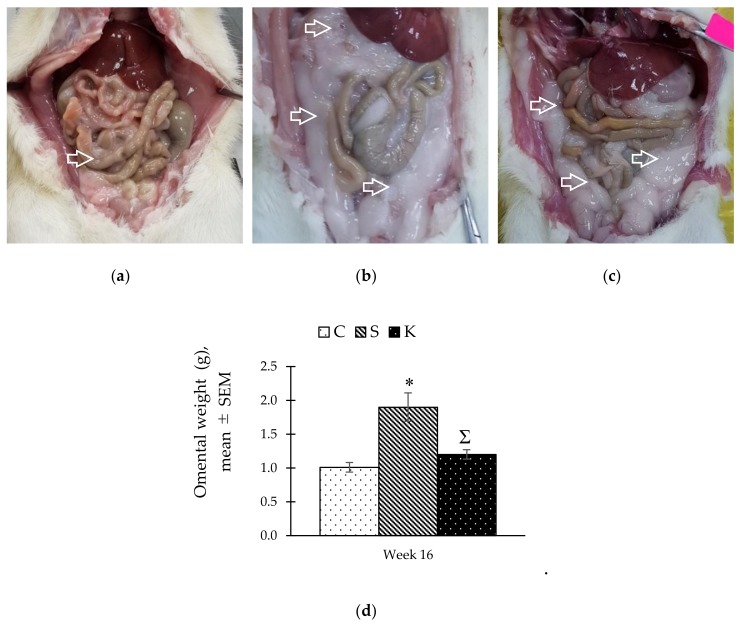
Comparison of visceral adipose tissue deposition in (**a**) C, (**b**) S, and (**c**) K rats. The bar chart in (**d**) shows the omental weight. Arrows represent visceral adipose tissue. * Significant difference compared to C, *p* < 0.05. ^Ʃ^ Significant difference compared to S, *p* < 0.05. The error bars represent the standard error of mean with *n* = 6 in each group. Abbreviation: C (control), S (HCHF diet-induced MetS), K (MetS + Kelulut honey).

In-situ observation upon longitudinal incision of the abdominal wall showed that the S group had the most abundant intraabdominal adipose tissue deposition which was reduced in the K group by KH supplementation. (Figure 6a–c). Consistent with these findings, the S group had the heaviest gross omental fat mass compared with the C rats (1.90 ± 0.21 g vs. 1.01 ± 0.07 g, *p* = 0.017). The omental fat mass was reduced significantly in the K group by KH supplementation (1.20 ± 0.07 g, *p* = 0.047) (Figure 6d).

### 3.8. Changes in the Histomorphometry of the Visceral Adipose Tissue

**Figure 7:**
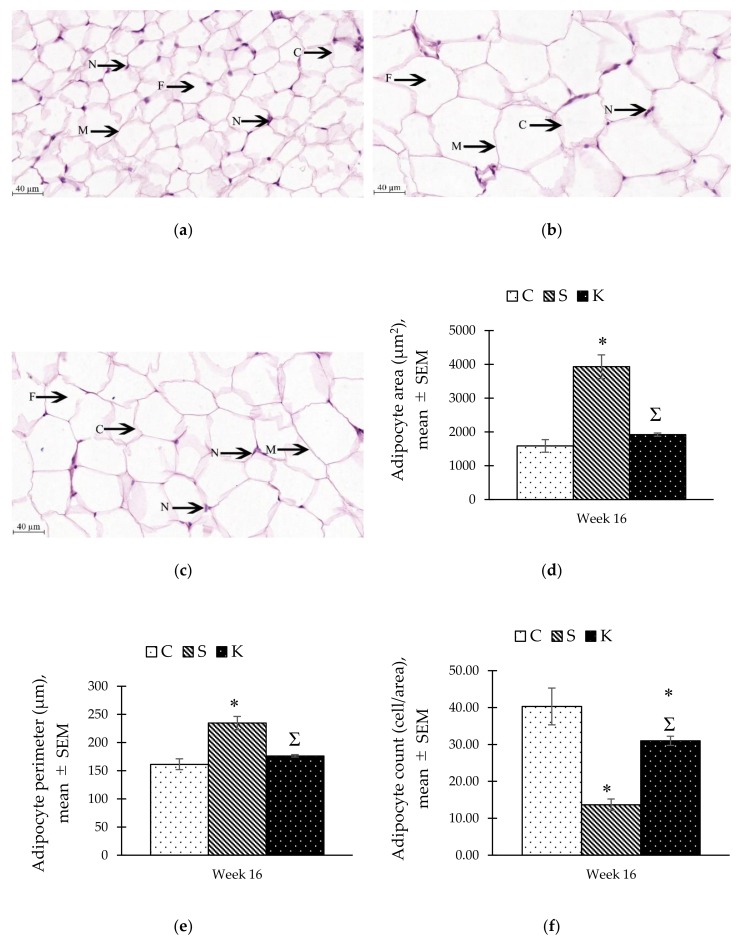
Photomicrograph of visceral adipose tissue in (**a**) C, (**b**) S, and (**c**) K rats under a light microscope with 400× total magnification. The size of adipocytes was indicated by the measurement of (**d**) area, and (**e**) perimeter. The bar chart in (**f**) shows the adipocyte count within the measuring area (350 µm × 250 µm). * Significant difference compared to C, *p* < 0.05. ^Ʃ^ Significant difference compared to S, *p* < 0.05. Abbreviation: C (cytoplasm), F (fat storage within the vacuole), N (nucleus), M (plasma membrane). The error bars represent the standard error of mean with *n* = 6 in each group. Abbreviation: C (control), S (HCHF diet-induced MetS), K (MetS + Kelulut honey).

Histological findings showed that MetS caused adipocytes hypertrophy (Figure 7a–c) which was confirmed by the measurements of the area (3,931.73 ± 348.79 µm^2^) and perimeter (234.75 ± 11.62 µm) of the adipocytes from the S group compared to the C group (1585.37 ± 187.05 µm^2^ and 161.52 ± 9.63 µm, respectively) (*p* < 0.05). Conversely, KH supplementation reduced these parameters significantly in the K group (1920.97 ± 45.49 µm^2^ and 176.45 ± 2.13 µm, respectively) (*p* < 0.05) to a level approaching the control (Figure 7d–e). Adipocyte count for the S group was significantly lower than the C group (13.67 ± 1.54 cell/area vs. 40.33 ± 4.98 cell/area, *p* < 0.001), while the K group had a significantly higher number of adipocytes than the S group (31.00 ± 1.26 cell/area, *p* < 0.05) (Figure 7f).

## 4. Discussion

The LC-MS profiling of KH in this study showed the presence of 4-hydroxyphenyl acetic acid, which is a major free-phenolic acids constituent in Malaysian stingless bee honey [30]. It possessed antioxidant property which has been shown to neutralize both reactive oxygen and nitrogen species [31,32] as well as antihypertensive effects as shown by Godos and colleagues [33]. Among 2044 adults of southern Italy, assessment of total phenolic intake showed that hydroxyphenyl acetic acid intake was inversely correlated with high blood pressure (highest vs. lowest quartile: OR 0.63, 95% CI 0.40–0.96). Additionally, coumaric and caffeic acids constituents in KH has been reported to have anti-obesity effect. Coumaric acid caused G_1_ cell cycle arrest in 3T3-L1 preadipocytes [34], whereas caffeic acid inhibited fatty acid synthase, which reduced serum TG and subsequently decreased visceral fat mass [35].

Induction of MetS in rodents through HCHF diet has been well-established by previous studies [28,29]. It mimics the dietary habit of the Western population with high consumption of saturated fat and wide use of refined sugar as sweeteners in beverages [36]. In this study, HCHF diet for 16 weeks successfully induced MetS by causing central obesity, high blood pressure, and hypertriglyceridemia in rats, which fulfilled at least three criteria put forward by the harmonized definitions of MetS [1]. The study also showed that KH supplementation significantly attenuated the metabolic changes caused by MetS in rats.

Rats fed with the HCHF diet displayed lower body weight compared to control. This could be attributed to the decrease of lean mass, which is an indirect measurement of muscle mass [37], despite having an increase in the fat mass. Adequate dietary protein intake has been linked to prevent muscle mass loss [38]. The protein content of rat chow is 21–23% [39], followed by sweetened condensed milk (7%) and ghee (0.1%). Control rats received 21–23% protein while MetS-induced rats only received 6.1–6.4% protein from the rat chow, sweetened condensed milk and ghee constituents of the HCHF diet. This might explain the marked loss of lean mass in both S and H groups. Consistent with our findings, Ribeiro and colleagues reported that rats receiving lower amount of protein in their diet had a significantly lower gastrocnemius muscle mass compared to higher protein diet (1.38 ± 0.14 vs. 1.63 ± 0.17 g, *p* < 0.05) [40].

In this study, HCHF diet did not induce hyperglycemia and hyperinsulinemia in the rats. The administration of KH also did not exert any effect on both parameters. However, MetS could occur in the absence of impaired glucose levels. A previous study reported that MetS was detected in normoglycemic human subjects. From a total of 1222 individuals, 27.6% of the 555 normoglycemia subjects were diagnosed with MetS when they fulfilled other diagnostic criteria of ATP III such as high serum TG, low serum HDL, and high WC readings [41]. We hypothesize that the insulin resistance was still in its early stages and may take more than 16 weeks of HCHF diet to produce significant changes in our study.

Elevated serum TG in rats fed with HCHF diet could be explained by the high-fructose and high-fat content in the diet [42,43,44]. A meta-analysis study showed that 10-21% (w/v) fructose intake by rats was directly proportional to the increase in TG (standard mean: 1.87, 95% confidence interval: 1.39–2.34, *p* < 0.0001) [45]. Fructose stimulates lipogenesis through several mechanisms including absorption through insulin-independent GLUT-5 [46], metabolism by the hepatic fructokinase which required lower fructose concentration in the plasma [47], as well as the ability of fructose to prevent the inhibition of pyruvate dehydrogenase that will drive acetyl-CoA synthesis to produce lipid-like TG [48]. Ultimately, excessive fructose and lipid intake from the diet will be transported through the circulation via lipoprotein such as LDL to the adipocytes for storage in the form of TG [49]. The hypotriglyceridemic effect by KH was similar to the findings by Aziz and colleagues [14]. The administration of stingless bee honey from *Geniotrigona thoracica* species reduced serum TG levels significantly in streptozotocin-nicotinamide-induced male diabetic rats. The hypotriglyceridemic effect was not only shown by the KH but also honey from other bee species [17,50]. Non-digestible oligosaccharides such as fructo-oligosaccharides (FOS) that were found in honey may be responsible for this effect [51,52]. Nakamura and colleagues reported that FOS administration in lipid emulsions significantly reduced serum TG in mice compared to the control by preventing dietary fat absorption from the small intestine [53]. Apart from that, the hypotriglyceridemic effect of KH in this study might also be contributed by coumaric and caffeic acids which were detected via LC-MS as mentioned previously.

Serum TC was found to be unchanged by HCHF and honey administration in the current study. This may be explained by the rats’ natural resistance against hypercholesterolemia. A prior study reported that five of nine rat strains maintained normal levels of serum TC after cholesterol challenge (addition of 1% cholesterol in diet) for 21 days [54]. The five strains, including Wistar, were observed to have a higher basal activity of hepatic β-hydroxy-β-methylglutaryl-CoA (HMG-CoA) reductase. Thus, dietary cholesterol imposed a greater inhibition of the HMG-CoA reductase activity, decreasing the de novo cholesterol synthesis and prevent the further rise of serum TC. On the contrary, the degree of HMG-CoA reductase inhibition by dietary cholesterol was minimal in rat strains with a lower basal activity of the enzyme. This led to increased serum TC upon cholesterol challenge.

Elevated blood pressure was observed in MetS rat models in several studies which was consistent with our findings [29,55]. It was closely related to the high oxidative stress as shown by elevated superoxide levels in erythrocytes and malondialdehyde in plasma [29]. Oxidative stress caused a reduction of nitric oxide bioavailability leading to subsequent vasoconstriction and increased blood pressure [56]. In addition, the HCHF diet used in the current study has been reported to damage the thoracic aorta endothelial function, thus reducing the vaso-relaxation rate, resulting in hypertension [57]. The ability of KH to lower blood pressure may lie on its antioxidant properties [20]. Honey supplementation in spontaneously hypertensive rats at the dose of 1 g/kg/day was shown to lower oxidative stress in their kidneys by upregulating the Nrf2 mRNA protein expression that activated the synthesis of antioxidant enzymes such as catalase and glutathione-S-transferase [16]. These enzymes neutralized the reactive oxygen species in the kidneys which ultimately normalized the blood pressure [58].

In the current study, the HCHF diet caused central obesity in rats as evidenced by a significant increase in body fat percentage compared to the control. Rats receiving KH supplementation had lower body fat percentage and omental fat compared to the unsupplemented MetS-induced rats. This observation was in agreement with earlier studies, in which rats receiving long-term honey treatment experienced a significantly lower body fat percentage compared with the sucrose-fed group [59]. Central obesity was shown by previous studies to cause a low-grade systemic inflammation as evidenced by the high level of inflammatory markers in the blood [60,61,62]. The anti-obesity effect of KH may be contributed by its polyphenolic content which possesses both anti-inflammatory and anti-obesity properties [63,64,65].

Adipose tissue can undergo hypertrophy and hyperplasia when exposed to exogenous stimuli like dietary components [66]. In obesity, adipocyte hypertrophy is a major contributor to the increase in fat mass as shown in this study. The hypertrophied adipocytes in rats receiving HCHF diet caused the cells to fill in more space in the measured area, thereby reducing the adipocytes counts significantly compared to the control. These findings were similar to a study by Mammikutty et al. [67]. In contrast, adipocyte hyperplasia did not significantly contribute to the fat mass as the newly formed cells had limited space for fat storage [68]. Furthermore, adipocyte hyperplasia only occurred during childhood and pre-puberty [69]. The rate of adipocyte proliferation decreased during adolescence and remained stable throughout adulthood [70]. The anti-obesity effects of KH were conferred by reducing adipocyte hypertrophy which was in agreement with prior studies [18,71].

To the best of our knowledge, this is the first study that reports the beneficial effects of KH from the *H. itama* stingless honey bee species against MetS. However, the mechanism of KH in suppressing central obesity, high blood pressure, as well as hypertriglyceridemia in MetS were unknown. These research gaps should be bridged in future studies.

## 5. Conclusions

The HCHF diet induces MetS in rats by elevating the body fat percentage, omental fat mass, serum TG, SBP, and DBP, as well as causing adipocyte hypertrophy which are reversed by KH supplementation. These positive findings provide the basis for future KH research to investigate its potential use against MetS in humans and its molecular mechanisms in alleviating the disease.

## Figures and Tables

**Table 1:** LC-MS profiling of Kelulut honey from *Heterotrigona itama* stingless bee.

No.	Mass (Da)	Retention Time (min.)	Compound Name	Molecular Formula
1.	151.3016	2.997	4-Hydroxyphenyl Acetic Acid	C_8_H_7_O_3_
2.	474.3035	3.278	Caffeic Acid Derivative	C_9_H_8_O_4_
3.	522.9860	2.828	Caffeoyl Glucose Derivative	C_15_H_18_O_9_
4.	503.0967	2.379	Caffeoyl Hexoside Derivative	C_15_H_17_O_9_
5.	162.6028	2.773	Coumaric Acid	C_9_H_8_O_3_
6.	195.3490	2.941	Gluconic Acid	C_6_H_12_O_7_
7.	143.6920	3.390	Kynurenic Acid Derivative	C_10_H_7_NO_3_
8.	271.4930	1.708	Pinobanksin	C_15_H_12_O_5_
9.	191.0423	1.875	Quinic Acid	C_7_H_12_O_6_
10.	357.1258	6.689	Niazimicin	C_16_H_23_NO_6_S
11.	488.1492	4.064	Bisosthenon B	C_28_H_24_O_8_
12.	384.1932	4.727	(6β,7α,12β,13β)-7-Hydroxy-11,16-dioxo-8,14-Apianadien-22,6-Olide	C_23_H_28_O_5_
13.	365.2007	8.411	Aegle Marmelos Alkaloid C	C_23_H_27_NO_3_
14.	341.0460	11.911	7-chloro-6-Demethylcepharadione B	C_18_H_12_ClNO_4_
15.	117.0415	9.870	N-Acetylglycine	C_4_H_7_NO_3_
16.	426.3835	10.650	Lanosterol	C_30_H_50_O
